# Poster Session II - A233 DIETARY ENHANCEMENT OF MICROBIAL TRYPTOPHAN METABOLISM RESTORES AHR SIGNALLING AND REDUCES COLITIS

**DOI:** 10.1093/jcag/gwaf042.233

**Published:** 2026-02-13

**Authors:** L Rondeau, B Barbosa da, Luz Confetti, P Muppidi, R Dang, X Wang, A Caminero

**Affiliations:** Medicine, McMaster University, Hamilton, ON, Canada; Medicine, McMaster University, Hamilton, ON, Canada; Medicine, McMaster University, Hamilton, ON, Canada; McMaster University, Hamilton, ON, Canada; Medicine, McMaster University, Hamilton, ON, Canada; Medicine, McMaster University, Hamilton, ON, Canada

## Abstract

**Background:**

Intestinal microbiota, diet, and the immune system contribute to the development of inflammatory bowel diseases (IBD). The aryl hydrocarbon receptor (AhR) is a critical regulator of intestinal immunity and barrier function, activated by microbial and host tryptophan (Trp) metabolites. IBD patients in large cohorts and in our own studies show reduced bacterial Trp metabolism genes and metabolites, which is associated with downregulation of colonic AhR activation, but the contribution of microbiota to this phenotype is unclear. Enhancing Trp metabolism through diet/probiotics may offer a therapeutic strategy to reduce inflammation in IBD.

**Aims:**

To investigate the impact of diet-microbe interventions on AhR activation in mice colonized with IBD and mouse microbiota with impaired Trp metabolism.

**Methods:**

Germ-free C57BL/6 mice were colonized with microbiota from human healthy controls (HC) or IBD patients, and specific pathogen free (SPF) or a minimal microbiota composed of 8 species (MM). Mice were provided a high Trp (HT; 1% Trp) or control diet (CD; 0.14% Trp). Subsets of mice received probiotic *Clostridium sporogenes* with Trp metabolism genes. Colitis was induced in mice using dextran sulfate sodium (DSS; 2.5% w/v), 2,4,6-trinitrobenzenesulfonic acid (TNBS; 2% w/v), and IL-10^-/-^ models. AhR antagonist (CH223191) or vehicle were provided daily during colitis. Trp metabolites quantified by LC-MS. AhR activation was quantified by AhR reporter and RT-qPCR. Histologic analysis of distal colon, clinical scores, intestinal permeability (Ussing chambers), and inflammatory genes (Nanostring) were assessed.

**Results:**

Humanized mice colonized with IBD microbiota had reduced Trp metabolism vs HC. MM microbiota mice had reduced Trp metabolism vs SPF. Reduced Trp metabolism associated with exacerbated colitis severity. HT diet supplementation in IBD humanized mice increased Trp metabolism, activated AhR, and reduced DSS, TNBS, and IL-10^-/-^ colitis. Probiotic supplementation was required with HT diet to achieve clinical colitis alleviation in mice with severe impairment of AhR signalling.

**Conclusions:**

Transfer of AhR activation from humans to mice by microbiota transplant indicates that AhR activation is microbiota dependent. Impaired host/microbial Trp metabolism increases susceptibility to colitis. Trp supplementation and microbial interventions offer an approach to restore AhR signalling and reduce intestinal inflammation.

**Funding Agencies:**

CAG, CCC, CIHR

